# Characterization of mouse neuro‐urological dynamics in a novel decerebrate arterially perfused mouse (DAPM) preparation

**DOI:** 10.1002/nau.23471

**Published:** 2018-01-15

**Authors:** Hiroki Ito, Marcus J. Drake, Christopher H. Fry, Anthony J. Kanai, Anthony E. Pickering

**Affiliations:** ^1^ School of Physiology, Pharmacology and Neuroscience, Faculty of Biomedical Sciences University of Bristol Bristol United Kingdom; ^2^ Department of Pharmacology and Chemical Biology University of Pittsburgh Pittsburgh Pennsylvania

**Keywords:** external urethral sphincter, lower urinary tract, micturition, mouse, neural control

## Abstract

**Aim:**

To develop the decerebrate arterially perfused mouse (DAPM) preparation, a novel voiding model of the lower urinary tract (LUT) that enables in vitro‐like access with in vivo‐like neural connectivity.

**Methods:**

Adult male mice were decerebrated and arterially perfused with a carbogenated, Ringer's solution to establish the DAPM. To allow distinction between central and peripheral actions of interventions, experiments were conducted in both the DAPM and in a “pithed” DAPM which has no brainstem or spinal cord control.

**Results:**

Functional micturition cycles were observed in response to bladder filling. During each void, the bladder showed strong contractions and the external urethral sphincter (EUS) showed bursting activity. Both the frequency and amplitude of non‐voiding contractions (NVCs) in DAPM and putative micromotions (pMM) in pithed DAPM increased with bladder filling. Vasopressin (>400 pM) caused dyssynergy of the LUT resulting in retention in DAPM as it increased tonic EUS activity and basal bladder pressure in a dose‐dependent manner (basal pressure increase also noted in pithed DAPM). Both neuromuscular blockade (vecuronium) and autonomic ganglion blockade (hexamethonium), initially caused incomplete voiding, and both drugs eventually stopped voiding in DAPM. Intravesical acetic acid (0.2%) decreased the micturition interval. Recordings from the pelvic nerve in the pithed DAPM showed bladder distention‐induced activity in the non‐noxious range which was associated with pMM.

**Conclusions:**

This study demonstrates the utility of the DAPM which allows a detailed characterization of LUT function in mice.

## 1 INTRODUCTION

The micturition cycle consists of two‐phases of bladder activity: filling and voiding. This is achieved by the co‐ordinated action of both the autonomic and somatic motor system. A spinal‐brainstem‐spinal loop is the core circuit controlling micturition including the periaqueductal gray and pontine micturition center (Barrington's nucleus). This circuit responds to afferent inputs from the bladder to generate voiding.^1^, ^2^ However, the neural mechanisms that generate and regulate the phases of micturition^1^, ^2^ have not been fully elucidated in man nor in animal models.

Rodents are now the most common model species for neuro‐urological investigations and have been widely adopted for studies of the lower urinary tract (LUT). Previously, we have reported a decerebrate, arterially perfused rat (DAPR) preparation which exhibited strong and consistent filling and voiding responses.^3^, ^4^ This preparation permits the simultaneous measurement of bladder pressure, external urethral sphincter‐electromyogram (EUS‐EMG) and nerve recordings from pelvic or pudendal nerves, as well as recordings of the phrenic nerve, electrocardiogram, and arterial pressure.^4^ However, a drawback of DAPR for some types of studies was the need to use young rats primarily due to the challenge of establishing adequate tissue perfusion in larger animals, which limited preparation viability.

The mouse is increasingly used in neuroscience investigations because of its genetic tractability and the ability to study the influence of precise molecular interventions at a circuit level, however, the study of LUT control mechanisms in the mouse is in its infancy.^5^ Therefore, to take advantage of this toolbox and tractability and to overcome the size/age limitations of DAPR we have developed a decerebrate arterially perfused mouse (DAPM) preparation that allows the complete micturition cycle of adult animals to be investigated. We show this approach can be used to fill some of the current gaps in our knowledge regarding the dynamics of murine neuro‐urology^5^ by improving access to both the LUT and the neural elements and permitting vitro‐like access for like application of pharmacological agents without the requirement for anaesthesia.

## 2 MATERIALS AND METHODS

All experiments conformed to the UK Animals (Scientific Procedures) Act 1986 and were approved by the University of Bristol local Ethical Review Panel. These methods are based on those previously described for the DAPR.^3^, ^6^ In the following description an emphasis is placed on those aspects of the technique that are different in the mouse.

### 2.1 Set‐up of decerebrate arterially perfused mouse (DAPM) preparation

Male mice (CD1, 34‐36 g, *n* = 67 (50 DAPM and 17 pithed DAPM)) were used in these experiments (note the DAPM could also be successfully established with adult female mice). Mice received heparin (50 IU, ip) to prevent clot formation twenty minutes before being anaesthetized with isoflurane (2%) until loss of paw withdrawal reflex. After a midline laparotomy, the stomach and intestine were vascularly isolated and removed (leaving spleen, duodenum, initial part of jejunum, and sigmoid colon). The ureters were cut bilaterally and ligated distally to prevent bladder filling. The thoracic cavity was opened via a midline sternotomy, causing respiratory arrest, and gaseous anaesthesia was discontinued (without gas exchange the deep level of anaesthesia would be maintained). The animal was cooled by immersion into modified Ringer's solution at 5‐6°C on ice (composition below). After craniotomy, the mouse was decerebrated, by aspiration, at the pre‐collicular level. At this point the animal terminally exsanguinated and was formally considered dead.

The preparation was skinned then pinned to a Sylgard‐covered dissecting dish on ice. The left phrenic nerve was identified and the lungs/diaphragm removed. An incision was made at the cardiac apex into the left ventricular cavity for the perfusion cannula. The right atrium and inferior vena cava were incised to prevent venous hypertension during arterial perfusion.

**Figure 1:**
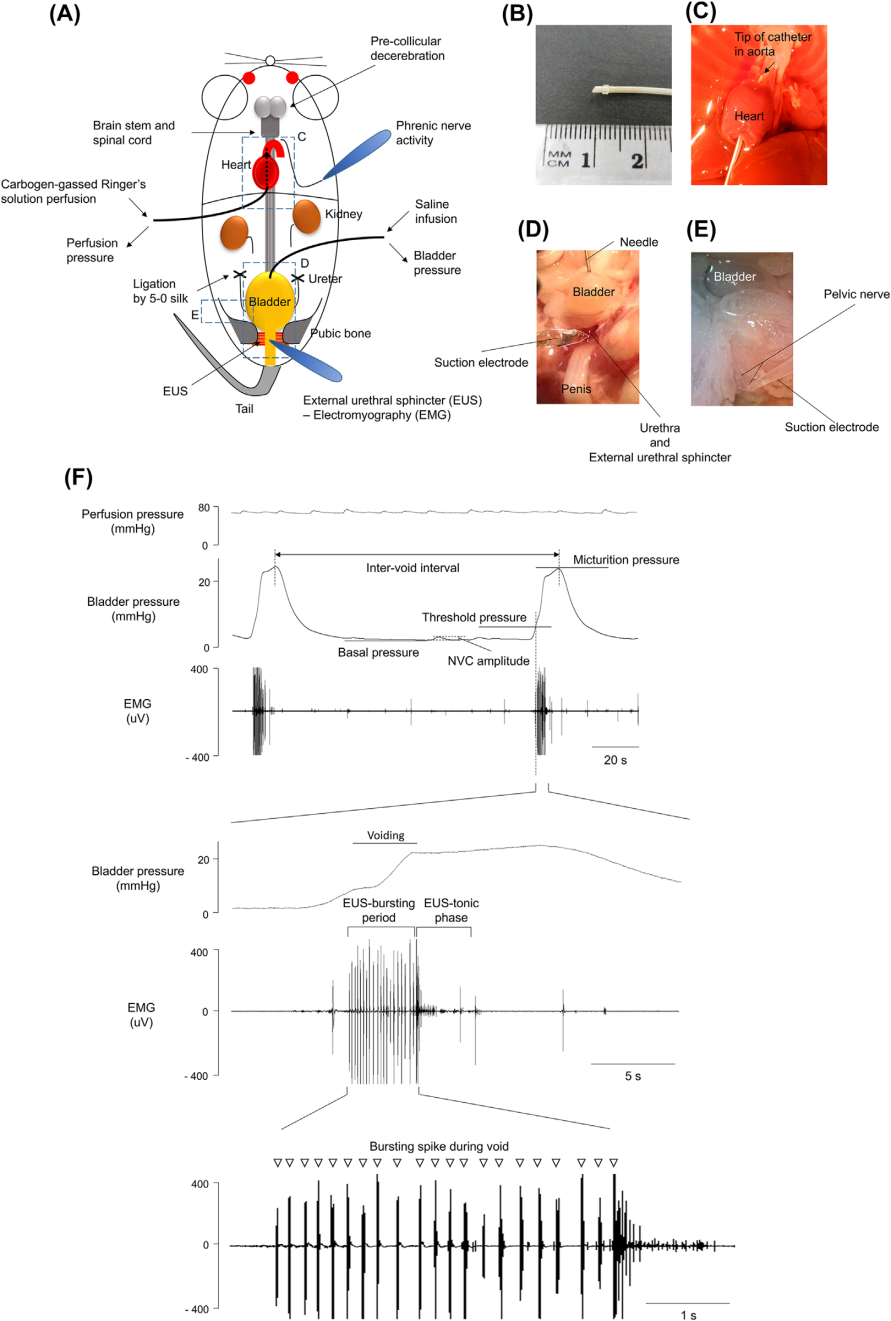
Schematic of decerebrate arterially perfused mouse (DAPM) in situ preparation for bladder studies (A). The perfusion cannula is shown with a knuckle at the tip to secure its placement in the aortic arch (B). The cannula is inserted under direct vision through the left ventricle (C) and pushed through the aortic valve until the knuckle fits through the aortic valve tightly and the tip is visible in the proximal aorta. (D) Lower urinary tract showing a needle inserted into the bladder dome allowing infusion of fluid and monitoring of intravesical pressure. A suction electrode enabled simultaneous EUS‐EMG activity recording. (E) Pelvic nerve of the bladder held in a suction electrode for recording of afferent activity. (F) Consecutive micturition cycles recorded with EUS‐EMG activity. Expanded time scale shown below with EUS bursting activity during voiding. The following micturition parameters (Figure 1F) were measured (averaged over at least three voiding cycles): 
 Baseline was taken as the lowest bladder pressure reached immediately following a void. Voiding threshold was the bladder pressure when the EUS‐EMG changes its activity to bursting, indicating the initiation of voiding. Micturition pressure was the absolute value of the peak bladder pressure achieved during voiding (bursting phase of the EUS‐EMG). Non‐voiding contractions (NVCs) were identified as discrete increases in bladder pressure (>1 mmHg) observed during the filling phase in voiding preparations. In preparations without voiding (ie, pithed DAPM) the small rhythmical pressure fluctuations with amplitude of more than 0.4 mmHg were termed putative micromotions (pMM). Bladder compliance was defined as bladder capacity/(threshold—basal pressure) (μL/ΔmmHg) during filling at a rate of 25 μL/min

The preparation was transferred to the recording chamber and a flushed double lumen cannula (Ø 1.2 mm, Argyle Covidien, MA) was inserted into the ascending aorta through the left ventricle. The end of the catheter was passed through the aortic valve and was held in the ascending aorta by a wedge‐shaped sleeve just proximal to the cannula tip (Figure 1A‐C). The preparation was perfused with carbogen‐gassed Ringer's containing an osmotic agent, Ficoll‐70 (1.25% Sigma). The heated perfusate (31°C) was pumped (15‐20 mL/min, Watson‐Marlow 505D, UK) from a reservoir flask through bubble traps and a particle filter (25 µm screen, Millipore). Perfusate was recycled from the recording chamber to the reservoir. Aortic perfusion pressure was monitored via the second lumen of the cannula. Once perfusate flow was initiated the heart resumed beating. Rhythmic respiratory movements of the chest wall were seen within 5 min, indicating the return of brainstem activity, typically as perfusion pressure reached 50‐60 mmHg. However, at this perfusion pressure, coordinated bladder‐EUS control was often not observed and it typically required ∼65‐70 mmHg for voiding to commence, likely reflecting the need to perfuse the distal spinal cord adequately, as noted in the DAPR.^3^ This was achieved by care with arterial haemostasis during surgical preparation. If the initial perfusion pressure remained low then it could be increased by addition of Angiotensin II to the perfusate, however, overzealous use of Angiotensin II (at doses of >100 pM) could cause deterioration of the eupnoeic respiratory pattern, perhaps because of excessive vasoconstriction.

A glass suction electrode was used to record the left phrenic nerve whose activity (PNA) was AC amplified (5‐10 k), band pass filtered (100 Hz to 3 kHz) and digitized at 10 KHz. The preparation was not paralyzed with muscle relaxant (except where specified), in order to retain EUS function. Therefore, the preparation displayed respiratory movement throughout the experiments and this, alongside the eupnoeic pattern of phrenic nerve activity, gave a continuous index of viability.

### 2.2 Lower urinary tract recordings

The pubic symphysis was cut in the midline to allow access to the EUS. A glass suction electrode (tip diameter ∼200 µm) was placed on the proximal sphincter slightly lateral to the midline, directly below the bladder neck to record EUS‐EMG (Figure 1D). A reference Ag/AgCl wire electrode was fixed close to the tip. A 27G needle was inserted into the bladder dome and connected via saline‐filled tubing to a pressure transducer and a syringe pump (Genie, Kent Scientific, Torrington, CT) allowing infusion of 0.9% saline. The infusion rate was 25 μL/min (unless otherwise stated), as used in previous mouse urodynamic studies.^5^ To test the experimental utility of DAPM for the assessment of peripheral bladder sensitization, the effect of intravesical acetic acid (0.2%) was evaluated.

### 2.3 “Pithed” DAPM

To investigate the peripheral effects of drugs by excluding any potential effects on CNS, we established the DAPM and then transected the cord at the medullo‐spinal junction to allow removal of the brain stem and “pithed” the spinal cord with a blunt wire. The ablation was confirmed by a total loss of phrenic nerve activity and of respiratory/pinch‐evoked movements. Additionally, the micturition cycle was lost and the LUT became incontinent. Therefore, the urethra was clamped to allow measures of filling pressure and compliance. Saline was infused into the bladder to a maximum intra‐vesical pressure of 15 mmHg to avoid over‐distension. For recordings of pelvic nerve activity, the nerve was freed from surrounding tissue proximal to the major pelvic ganglion with fine forceps and then cut to allow access to the distal end with a suction electrode (Figure 1E).

### 2.4 Data acquisition

Perfusion pressure, phrenic nerve activity, ECG, bladder pressure, EUS‐EMG activity, and pelvic afferent neural activity were recorded using custom built AC amplifiers and transducers (built by Mr. Jeff Croker, University of Bristol). Signals were digitized using a micro1401 interface (Cambridge Electronic Design (CED), Cambridge, UK) to a computer running Spike2 software (version 7, CED).

### 2.5 Analysis

Analysis was conducted offline, using Spike2 software and the Statistical Package for Social Sciences, version 22 (SPSS Inc., Chicago, IL). Statistical testing employed parametric or non‐parametric tests as appropriate given the distribution of the sampled parameters as assessed by Shapiro‐Wilk normality test. All values are expressed as the mean ± standard error or median (25‐75th percentiles) and *n* = number of preparations. The threshold for statistical significance was *P* < 0.05.

### 2.6 Drugs and solutions

The composition of Ringer's was NaCl (125 mM), NaHCO_3_ (24 mM), KCl (3 mM), CaCl_2_ (2.5 mM), MgSO_4_ (1.25 mM), KH_2_PO_4_ (1.25 mM); Glucose (10 mM) pH 7.35‐7.4 after carbogenation (95% O_2_/5% CO_2_). Ficoll‐70 (1.25%) was added as an oncotic agent to the perfusate. Stock solutions of hexamethonium (330 mM), Angiotensin II (100 nM), and vasopressin (10 uM) were made in distilled water and kept frozen until the time of experiment, when they were diluted in Ringer's and perfused at the final concentration. All salts and drugs were from Sigma.

## 3 RESULTS

### 3.1 Preparation viability

After the initial optimization of preparation perfusion, an augmenting eupnoeic pattern of phrenic activity indicated healthy brainstem function^6^, ^7^ and typically lasted for periods of 3‐4 h. Respiratory movements of the thoracic cage, upper airway muscles, and motor responses to hind limb/tail pinch were seen throughout this phase.

### 3.2 Characteristics of fluid infusion‐evoked voids

Functional neural coordination of the bladder and EUS was clearly demonstrable in the preparation in response to intravesical infusion of saline (25 μL/min, Figure 1F). An initial series of experiments with direct bladder visualization indicated that voiding was complete without any residual urine (confirmed after each void by aspiration of the bladder contents, *n* = 5). During each void, the bladder pressure increased as the detrusor contracted and bursting activity of the EUS was observed. The effect of this bursting activity was visible as rhythmic contractions of the sphincter. Subsequently, a post‐void pressure increase was caused by isometric bladder contraction when the EUS ceased bursting and displayed tonic activity. The bladder pressure returned to baseline as the detrusor relaxed after the void. The mean voided volume was 100 ± 8 μL with an inter‐void interval of 240 ± 19 s (*n* = 12). The full profile of voiding parameters in the DAPM is shown in Table 1.

**Table 1:** Voiding parameters in decerebrate arterially perfused mouse (DAPM)

Cystometrogram	Mean ± SEM
Basal pressure	6.6 ± 0.4 mmHg
Threshold pressure	14.5 ± 0.9 mmHg
Micturition pressure	22.9 ± 1.5 mmHg
Infused volume	100.1 ± 7.9 μL
Inter‐void interval	240.3 ± 19.0 s
Bladder compliance	14.4 ± 2.1 μL/mmHg
Non‐voiding contraction frequency	1.5 ± 0.4/min
Non‐voiding contraction amplitude	3.1 ± 0.5 mmHg
**External Urethral Sphincter Electromyogram**	
Spike frequency within burst	4.7 ± 0.3 Hz
Duration of bursting activity per void	5.2 ± 0.8 s

Typically, the DAPM preparation exhibited voiding cycles for 1‐3 h (mean of 110 ± 10 min (*n* = 12)). The end of each experiment was signaled by the appearance of incomplete voiding, indicated by a gradual increase in the basal bladder pressure. This likely reflected impaired communication between brain stem and the autonomic and motor nuclei of the distal spinal cord, as there was still eupnoeic respiratory activity evident on the phrenic nerve (indicating intact brainstem communication with the cervical cord).

**Figure 2:**
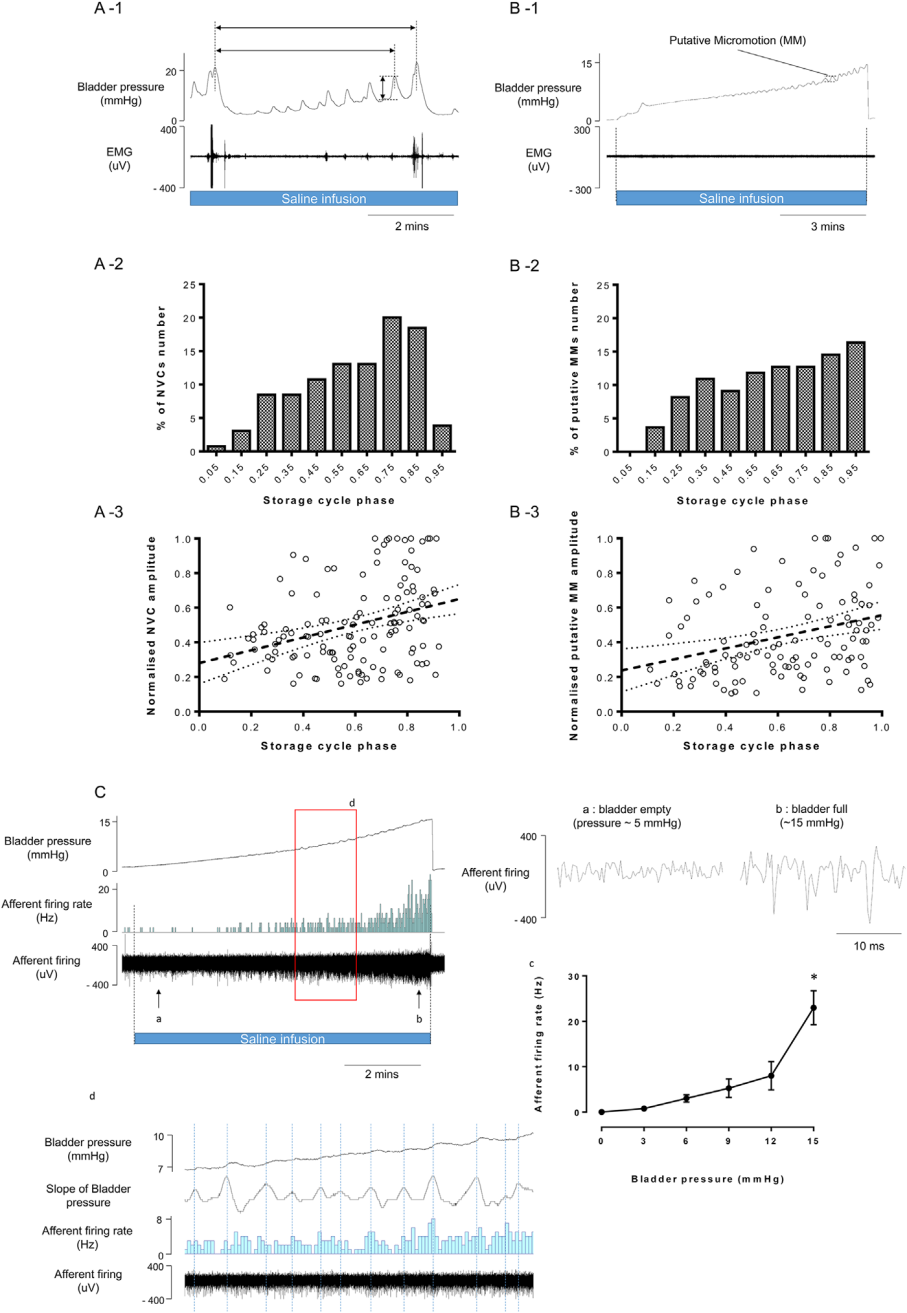
Relationship between NVCs, pMM, pelvic nerve activity and the phase of the micturition cycle. Both NVC amplitude and frequency increased with bladder filling (A‐1). The NVC amplitude and frequency showed a clear relationship to the phase of the micturition cycle (A‐2 and A‐3), normalized to the largest event/interval observed in each DAPM preparation, assessed over four micturition cycles, *n *= 7 mice). Similar relationship was found for pMM amplitude and frequency and micturition phase (*n* = 7 mice, B‐1, B‐2, and B‐3). Recording of pelvic afferent neural activity in pithed model (C) showing relationship to bladder pressure (Cc) and to pMM (Cd). * *P* < 0.05, significant difference compared to baseline with repeated measures ANOVA with Dunnett's post hoc test for multiple comparisons

Most preparations showed spontaneous NVCs during the storage phase with an average amplitude 2.6 ± 0.1 mmHg and frequency of 1.4 ± 0.1 per minute (total 130 NVCs from seven preparations analyzed in detail). Both the frequency and amplitude of NVCs increased with bladder filling (Spearman's coefficient = 0.333, *P* < 0.001, Figure 2A).

Rhythmic pressure waves that resulted from putative micromotions (pMM, as previously reported^8^) of the bladder wall were observed in the pithed model (Figure 2B). The amplitude of pMM (0.69 ± 0.04 mmHg, n = 236 from 7 mice) in the pithed model was 3‐4 fold smaller than amplitude of NVC in DAPM with an intact spinal cord and brainstem (*P* < 0.001). The frequency of pMM (1.7 ± 0.2 per minute (with bladder distensions up to 15 mmHg in 7 mice)) was similar to the frequency of NVCs in DAPM. Again, like NVCs, the pMM showed an increasing frequency and amplitude with bladder filling (Figures 2B‐1 and 2B‐2).

### 3.3 Actions of vasopressin on the LUT

In the DAPR, vasopressin (200‐400 pM) was used at the beginning of the preparation, as part of tuning, to increase the perfusion pressure and restore brainstem function.^3^ Accordingly, vasopressin was also initially trialed for tuning the DAPM, although it was found that higher doses of the pressor agent (>400 pM) were required to increase perfusion pressure above 60 mmHg. Despite achieving apparently good perfusion pressure and brainstem function (eupnoeic respiratory pattern), the DAPM commonly (>85% of the time, *n* = 23) did not exhibit an appropriate micturition cycle after application of vasopressin (2‐10 nM) and even at doses in the 200‐400 pM range normal micturition appeared to be impaired.

**Figure 3:**
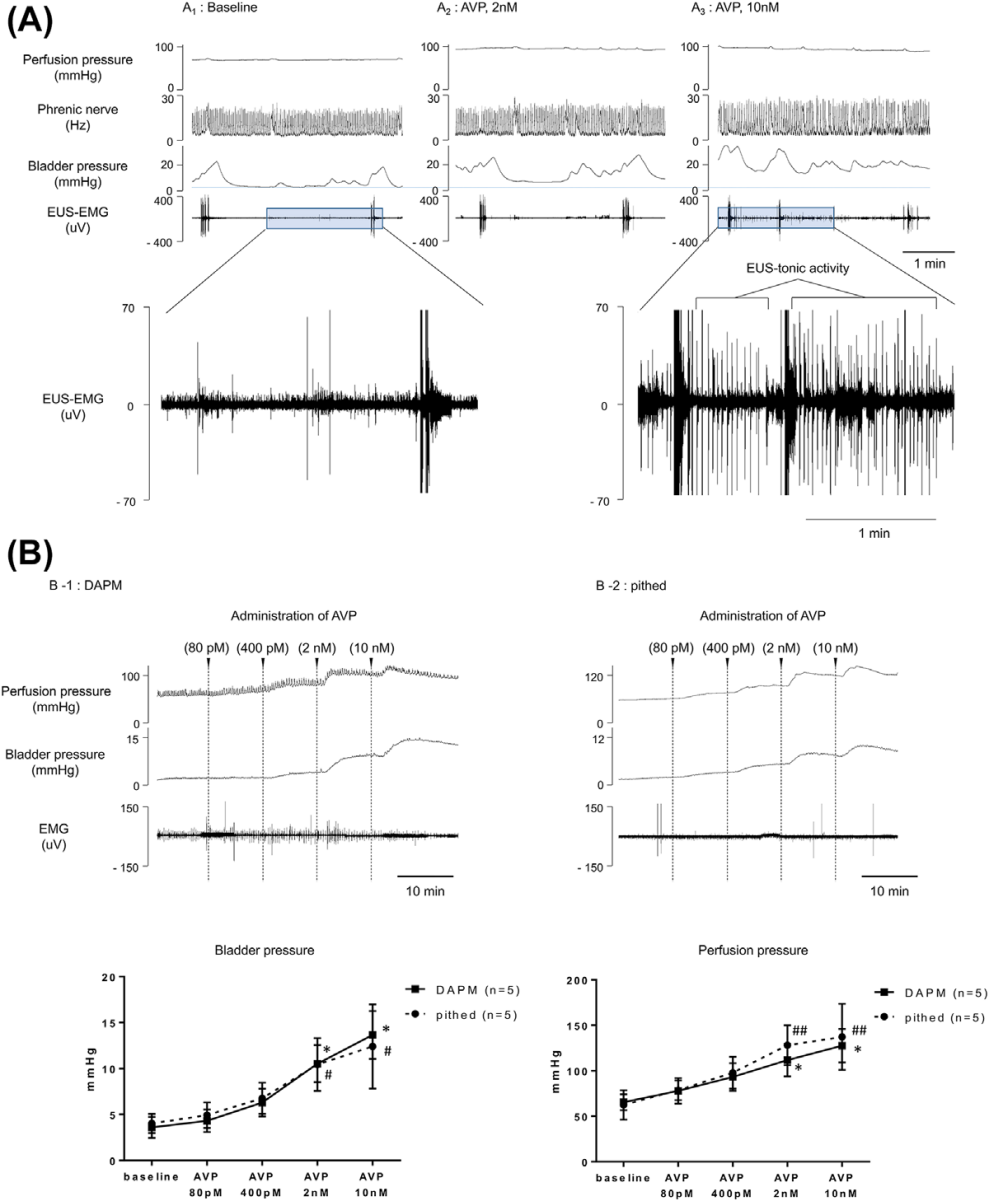
Effect of vasopressin on LUT function in the DAPM. (A) Representative recording of perfusion pressure, phrenic nerve activity, bladder pressure, and EUS‐EMG activity before and after administration of vasopressin (2 and 10 nM) showing the loss of bladder—sphincter co‐ordination manifesting as an increase in tonic activity on the EUS. (B) Direct effects of vasopressin (80 pM, 400 pM, 2 nM, and 10 nM) added to the perfusate on perfusion and bladder pressures in pithed and DAPM model (shown graphically below). *^, #^ *P* < 0.05, ^−^ *P* < 0.01, significant difference compared to baseline with repeated measures ANOVA and Dunnett's test for multiple comparisons (*—DAPM and ^#^—pithed DAPM)

This observation led us to assess whether vasopressin might be directly influencing bladder and urethral function in mice. Therefore, the DAPM was established by tuning with careful adjustment of flow alone to establish a eupnoeic breathing pattern and normal voiding. Subsequent addition of vasopressin to the perfusate (2 nM) increased the perfusion pressure (137.1% of control, Figure 3A). It also produced increases in both basal (121% of control) and micturition (127% of control) pressure (Figure 3A‐2). Further application of 10 nM vasopressin produced an increase in tonic EUS‐EMG activity, leading to urinary retention (Figure 3A‐3).

To test for effects of vasopressin on basal pressure (with a fill volume of 10 μL), incremental doses (0.08, 0.4, 2, and 10 nM) were added to the perfusate (at 10 min intervals). Vasopressin increased basal bladder pressure in a dose dependent manner (to 403 ± 46% of control with 10 nM of vasopressin, *n* = 5) (Figure 3B‐1). In the “pithed” DAPM (without CNS influence) vasopressin also increased bladder pressure (to 330 ± 34% of control, 10 nM vasopressin, *n* = 5) (Figure 3B‐2).

### 3.4 Autonomic and sacral motor outflows are required for active voiding and continence

**Figure 4:**
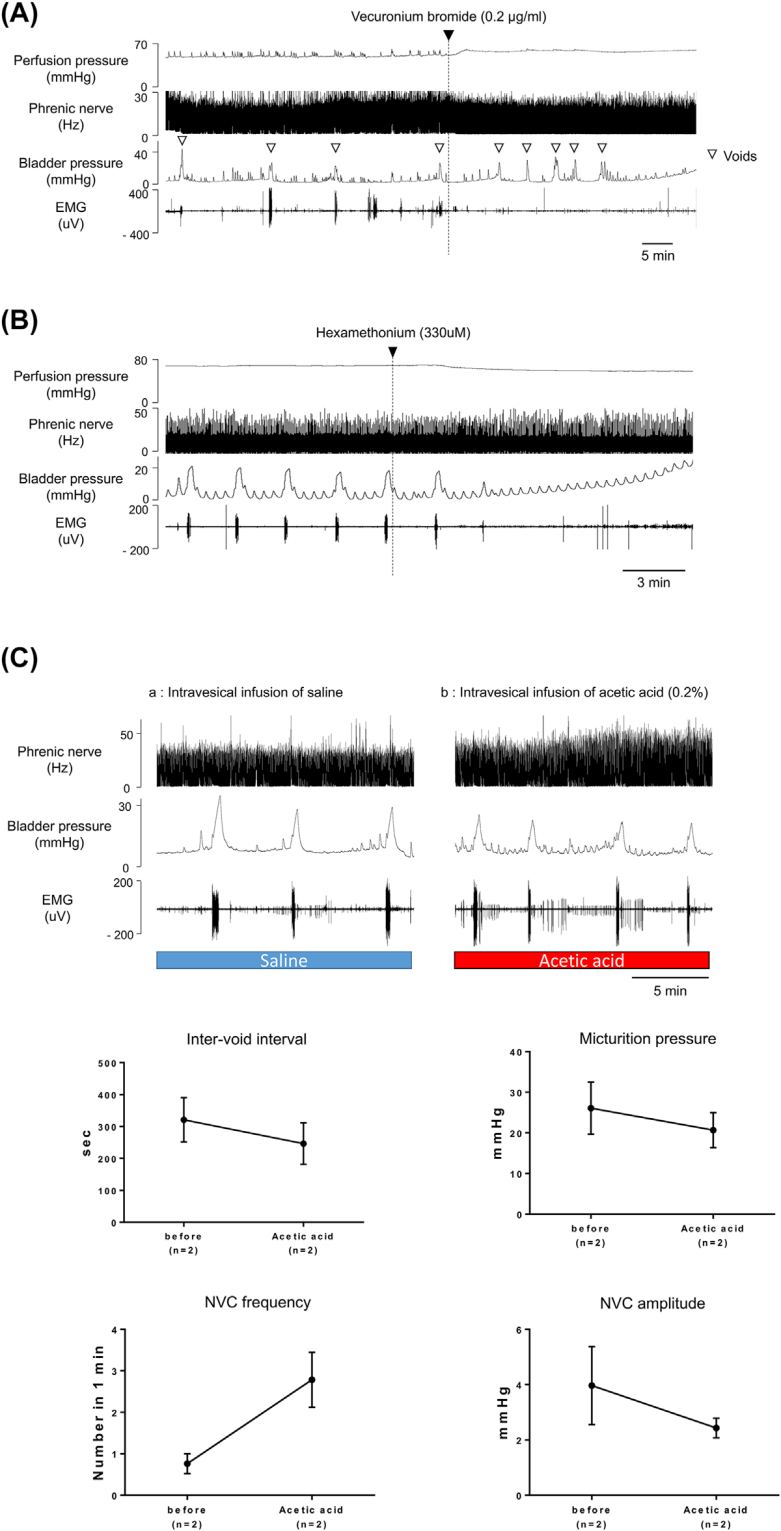
Urinary retention caused by administration of the neuromuscular blocker, vecuronium bromide (2 μg/mL) (A) and ganglion blocker, hexamethonium (330 μM) (B) to the perfusate. Effect of intravesical infusion of acetic acid (0.2%) on phrenic nerve activity, bladder pressure and EUS‐EMG activity (C‐A; Control, C‐B; After acetic acid)

Application of vecuronium (200 ng/mL), a neuromuscular blocking drug, caused the expected decrease in EUS‐EMG activity with a loss of bursting activity during voids. This caused incomplete voiding with a retained volume, indicating that the actively bursting EUS in the mouse appears to be important to facilitate urine passage through the sphincter. Further filling caused an increase in the bladder pressure, leading to passive leakage of fluid through the EUS, rendering the LUT incontinent (Figure 4A; *n* = 3).

Systemic administration of hexamethonium (330 μM after^3^), reduced arterial perfusion pressure (by 22.9 ± 2.6%, *n* = 4) indicating a loss of sympathetic tone consistent with autonomic ganglion blockade. This was not accompanied by a change in the eupnoeic PNA indicating that brainstem function was not compromised. The autonomic ganglion blockade initially caused incomplete voiding, with reduced peak voiding pressure and EUS activity and the preparation rapidly (within 3 min) stopped voiding. With further filling and an increase in bladder pressure then overflow urinary retention could be observed, that is, passive leakage of urine from the distended bladder (Figure 4B; *n* = 4).

### 3.5 Intravesical acetic acid

After obtaining baseline recordings of five voiding cycles acetic acid (0.2%) was infused into the bladder which decreased the micturition interval (to 75 ± 3.8% of control) and micturition pressure (to 75 ± 0.8% of control). The frequency of NVCs increased (to 385 ± 4.7% of control) and the amplitude of NVCs decreased (to 62 ± 1.3% of control). After infusion of acetic acid, the pattern of EUS‐EMG changed to exhibit phasic activity even when the bladder was almost empty, and the frequency of phrenic nerve activity increased (to 132 ± 10.1% of control). (Figure 4C).

### 3.6 Suitability for pelvic nerve recordings

The pelvic nerve in the mouse is small and difficult to access so we tested the viability of recording from the nerve in the pithed DAPM. The nerve was straightforwardly identified, sectioned, and bipolar recordings made from the distal cut end. This showed bladder distention‐induced activity which increased exponentially over the range of pressures normally associated with the micturition cycle (Figure 2C). Intriguingly the pMM seen during bladder filling were associated with increases in afferent nerve activity.

## 4 DISCUSSION

We have developed and validated a novel in situ mouse model to investigate LUT function without the confounding effect of anaesthesia and with some of the advantages of the accessibility afforded by in vitro approaches. Previously, the DAPR has proven to be useful for the study of autonomic control and has enabled the completion of detailed LUT investigations.^3^, ^4^, ^6^ The DAPM allows straightforward access to the EUS and much of the LUT under direct vision. In addition, the actions of drugs, including those that would be toxic in in vivo, can be examined either systemically, locally, or intravesically.

### 4.1 Voiding characteristics of DAPM

The DAPM showed an intact micturition cycle of discrete, active filling and voiding phases, with excellent reproducibility. Continuous intravesical infusion triggered the coordinated active contraction of the bladder and EUS activity to enable voiding. The EUS EMG activity showed a bursting pattern during the void. A post‐void isovolumetric pressure increase was noted as the EUS closed (tonic EMG activity) while the detrusor remained contracted.

The DAPM showed a similar range of cystometrogram parameters to that observed previously in mouse studies (reviewed in^5^). The micturition pressure (23 mmHg) was within the range of previous values of in vivo ambulatory,^9^ urethane‐anaesthetized,^10^ and decerebrate mice,^11^ although several papers have reported higher micturition pressures between 25 and 45 mmHg in conscious mice.^5^ One possible reason for this difference may be that in the DAPM, the bladder is not influenced by the pressure transmitted from the abdominal wall (which has been opened) or it may relate to the absent volitional element provided by forebrain centers. It should be noted that the pressures generated in the DAPM were sufficient to completely void urine from the LUT.

### 4.2 Characteristics of mouse external urethral sphincter

The entire length of the EUS can be observed clearly in the DAPM allowing accurate placement of the electrode to reliably obtain EMG recordings. The functional examination of EUS is typically difficult in mouse, in part because of their body size.^12^ Therefore, the co‐ordinated activity of the bladder and urethra is poorly characterized in mice, in contrast to numerous studies in rats. The DAPM showed a similar pattern of EUS activity to rats^3^, ^4^, ^13^ with striking bursting activity of EUS during the void. There have been several recent reports recording EUS‐EMG in conscious, tethered mice.^11^, ^14^ These reports showed low‐amplitude tonic EUS‐EMG activity between voids which increased in amplitude during bladder contraction, falling only at the peak of the contraction, coincident with voiding.^11^, ^14^ These studies showed no EUS bursting activity. However, bursting activity has previously been noted in chronically implanted mice.^5^ One possible reason for this difference is the challenge of obtaining good signal to noise in tethered chronic recordings and challenges with identifying the EUS. As detailed, below we propose that the bursting activity is a fundamental physiological feature of voiding in the mouse. This may be required because of the local sphincter anatomy and flow dynamics in the mouse (and rat) where the small diameter and relatively long length of the ureter mean that voiding occurs by a different mechanism than in larger mammals.^15^

### 4.3 Neuromuscular blockade and autonomic ganglion blockade

Systemic administration of vecuronium and hexamethonium both compromised voiding. Vecuronium, as expected, decreased the electrical activity of the EUS, confirming its mediation by striated muscle. This reduced voiding efficiency—there were still active bladder contractions but these were unable to completely empty the bladder contents. This indicates that relaxation of external sphincter does not cause passive leakage of urine in the normal range of pressures seen in the mouse filling cycle. This also suggests that bursting activity of EUS is required for elimination of urine in mice. Hexamethonium also produced overflow incontinence by disruption of parasympathetic and sympathetic activity confirming that the voiding cycle in the DAPM requires active contributions from the autonomic nervous system.

### 4.4 Vasopressin induces urinary retention

In the DAPR, vasopressin was effective as a pressor without any obvious influence on the micturition cycle.^1^, ^3^ In the DAPM, however, vasopressin increased bladder pressure and impaired coordination with EUS activity, which induced urinary retention in most cases. It appears likely that vasopressin influences the bladder directly as we observed the same effects of vasopressin in the “pithed” DAPM preparation. It has been noted that vasopressin (via V1a receptors) contracts mouse internal urethral sphincter in vitro.^16^ We found higher doses of vasopressin increased phasic EUS‐EMG activity, which might also represent a direct action of vasopressin on the sphincter. Such an action to increase EUS sphincter tone may be responsible for the bladder‐sphincter dyssynergy.

### 4.5 Stimulation with acetic acid

Acetic acid infusion has been used as a model of peripheral bladder sensitization^17^ and showed a similar profile of action in the DAPM. Acetic acid also produced changes in respiratory pattern indicating that the activation of bladder afferents was signaled to a brainstem level. This indicates that the DAPM may be useful to investigate pathophysiological models of bladder disease including overactive bladder^17^ or bladder pain syndrome.

### 4.6 Subtracting central influences in the DAPM

The “pithed” DAPM preparation proved useful to contrast central versus peripheral mechanisms controlling the LUT, as noted earlier for vasopressin. Additionally, in the pithed preparation, pMM were observed whose characteristics were similar to NVCs in the DAPM as both showed increased amplitude and frequency with bladder filling, as previously noted in the anaesthetized Guinea‐pig.^18^ Recordings from the isolated bladder of Guinea‐pig suggested that spontaneous activity originates from both the bladder mucosa and detrusor but different pharmacological profiles,^19^ as yet the source of this activity is not defined in mice. Interestingly the pMM were sufficient to modulate pelvic afferent activity which could be also observed in ex vivo mouse model^20^; perhaps consistent with the idea that a spinal amplification mechanism is responsible for turning pMM into NVCs which had a larger amplitude. The pithed DAPM reliably allowed afferent recordings of pelvic nerve in mice whose activity increased exponentially in the normal range of micturition pressures (<15 mmHg), a lower range than that previously seen with in vitro recordings of mouse pelvic activity.^21^

## 5 SUMMARY

We report a novel in situ preparation, the DAPM, and demonstrate its utility for studies of the LUT, but we note that it is likely to be useful for studies of other viscera/locomotion and their nervous control. Particular advantages are the speed and ease of set up, lack of need for anaesthesia, and the straightforward access the urogenital organs including the bladder and urethra in the adult mouse. The preparations showed good experimental reproducibility increasing power to identify differences which meant that relatively low numbers of animals were required in each protocol. Potential drawbacks of the preparation include the duration of viability (up to 3 h). Preparation viability showed a strong temperature dependence (as we have previously noted for in situ preparations) and 31°C appeared an optimal compromise. Additionally, the loss of forebrain control centers that are known to regulate the volitional and behavioral control of voiding precludes analysis of their contribution.^22^ By way of compensation, the DAPM offers the potential to visualize bladder and urethra movements, which can be video captured allowing relation of electrophysiology to the bladder movement.^23^ The potential to combine with bladder sheet and Ca^2+^ imaging experiments^24^ or optogenetic approaches^25^ might bring new insights to the relationship of urothelial transmitters and central nervous system activity.

## CONFLICTS OF INTEREST

None.

## References

[1] Sasaki M . Role of Barrington's nucleus in micturition. J Comp Neurol. 2005; 493:21–26. 16255005 10.1002/cne.20719

[2] Drake MJ , Fowler CJ , Griffiths D , Mayer E , Paton JF , Birder L. Neural control of the lower urinary and gastrointestinal tracts: supraspinal CNS mechanisms. Neurourol Urodyn. 2010; 29:119–127. 20025025 10.1002/nau.20841

[3] Sadananda P , Drake MJ , Paton JF , Pickering AE. An exploration of the control of micturition using a novel in situ arterially perfused rat preparation. Front Neurosci. 2011; 5:62. 21625609 10.3389/fnins.2011.00062 PMC3097374

[4] Sadananda P , Drake MJ , Paton JF , Pickering AE. A functional analysis of the influence of beta3‐adrenoceptors on the rat micturition cycle. J Pharmacol Exp Ther. 2013; 347:506–515. 24008334 10.1124/jpet.113.207340 PMC3807064

[5] Ito H , Pickering AE , Igawa Y , Kanai AJ , Fry CH , Drake MJ. Muro‐neuro‐urodynamics; a review of the functional assessment of mouse lower urinary tract function. Front Physiol. 2017; 8:49. 28220079 10.3389/fphys.2017.00049 PMC5292568

[6] Pickering AE , Paton JF. A decerebrate, artificially‐perfused in situ preparation of rat: utility for the study of autonomic and nociceptive processing. J Neurosci Methods. 2006; 155:260–271. 16499970 10.1016/j.jneumeth.2006.01.011

[7] Paton JF . A working heart‐brainstem preparation of the mouse. J Neurosci Methods. 1996; 65:63–68. 8815310 10.1016/0165-0270(95)00147-6

[8] Drake MJ , Kanai A , Bijos DA , et al. The potential role of unregulated autonomous bladder micromotions in urinary storage and voiding dysfunction; overactive bladder and detrusor underactivity. BJU Int. 2017; 119:22–29. 27444952 10.1111/bju.13598 PMC5177525

[9] Aizawa N , Homma Y , Igawa Y. Influence of high fat diet feeding for 20 weeks on lower urinary tract function in mice. Low Urin Tract Symptoms. 2013; 5:101–108. 26663378 10.1111/j.1757-5672.2012.00172.x

[10] Boudes M , Uvin P , Kerselaers S , Vennekens R , Voets T , De Ridder D. Functional characterization of a chronic cyclophosphamide‐induced overactive bladder model in mice. Neurourol Urodyn. 2011; 30:1659–1665. 21717507 10.1002/nau.21180

[11] Kadekawa K , Yoshimura N , Majima T , et al. Characterization of bladder and external urethral activity in mice with or without spinal cord injury‐a comparison study with rats. Am J Physiol Regul Integr Comp Physiol. 2016; 310:R752–R758. 26818058 10.1152/ajpregu.00450.2015 PMC4867409

[12] Andersson KE , Soler R , Fullhase C. Rodent models for urodynamic investigation. Neurourol Urodyn. 2011; 30:636–646. 21661007 10.1002/nau.21108

[13] Walters RD , McMurray G , Brading AF. Comparison of the urethral properties of the female guinea pig and rat. Neurourol Urodyn. 2006; 25:62–69. 16224796 10.1002/nau.20194

[14] DePaul MA , Lin CY , Silver J , Lee YS. Peripheral nerve transplantation combined with acidic fibroblast growth factor and chondroitinase induces regeneration and improves urinary function in complete spinal cord transected adult mice. PLoS ONE. 2015; 10:e0139335. 26426529 10.1371/journal.pone.0139335 PMC4591338

[15] Yang PJ , Pham J , Choo J , Hu DL. Duration of urination does not change with body size. Proc Natl Acad Sci U S A. 2014; 111:11932–11937. 24969420 10.1073/pnas.1402289111 PMC4143032

[16] Zeng J , Ekman M , Grossi M , et al. Vasopressin‐induced mouse urethral contraction is modulated by caveolin‐1. Eur J Pharmacol. 2015; 750:59–65. 25637087 10.1016/j.ejphar.2015.01.029

[17] Yoshiyama M , Araki I , Kobayashi H , Zakoji H , Takeda M. Functional roles of TRPV1 channels in lower urinary tract irritated by acetic acid: in vivo evaluations of the sex difference in decerebrate unanesthetized mice. Am J Physiol Renal Physiol. 2010; 298:F1351–F1359. 20237234 10.1152/ajprenal.00695.2009

[18] Biallosterski BT , van Koeveringe GA , van Kerrebroeck PE , Gillespie JI , de Wachter SG. Nonvoiding activity of the guinea pig bladder. J Urol. 2011; 186:721–727. 21683402 10.1016/j.juro.2011.03.123

[19] Kushida N , Fry CH. On the origin of spontaneous activity in the bladder. BJU Int. 2016; 117:982–992. 26207752 10.1111/bju.13240

[20] Heppner TJ , Tykocki NR , Hill‐Eubanks D , Nelson MT. Transient contractions of urinary bladder smooth muscle are drivers of afferent nerve activity during filling. J Gen Physiol. 2016; 147:323–335. 26976828 10.1085/jgp.201511550 PMC4810069

[21] Daly D , Rong W , Chess‐Williams R , Chapple C , Grundy D. Bladder afferent sensitivity in wild‐type and TRPV1 knockout mice. J Physiol. 2007; 583:663–674. 17627983 10.1113/jphysiol.2007.139147 PMC2277033

[22] Hou XH , Hyun M , Taranda J , et al. Central control circuit for context‐dependent micturition. Cell. 2016; 167:73–86 e12. 27662084 10.1016/j.cell.2016.08.073 PMC6217838

[23] Kanai A , Zabbarova I , Ikeda Y , et al. Sophisticated models and methods for studying neurogenic bladder dysfunction. Neurourol Urodyn. 2011; 30:658–667. 21661010 10.1002/nau.21120 PMC4572846

[24] Ikeda Y , Zabbarova IV , Birder LA , et al. Botulinum neurotoxin serotype A suppresses neurotransmitter release from afferent as well as efferent nerves in the urinary bladder. Eur Urol. 2012; 62:1157–1164. 22480459 10.1016/j.eururo.2012.03.031 PMC5515465

[25] Park JH , Hong JK , Jang JY , et al. Optogenetic modulation of urinary bladder contraction for lower urinary tract dysfunction. Sci Rep. 2017; 7:40872. 28098199 10.1038/srep40872 PMC5241665

